# Spatial and Sex-Specific Variation in Growth of Albacore Tuna (*Thunnus alalunga*) across the South Pacific Ocean

**DOI:** 10.1371/journal.pone.0039318

**Published:** 2012-06-19

**Authors:** Ashley J. Williams, Jessica H. Farley, Simon D. Hoyle, Campbell R. Davies, Simon J. Nicol

**Affiliations:** 1 Oceanic Fisheries Programme, Secretariat of the Pacific Community, Noumea, New Caledonia; 2 CSIRO Marine and Atmospheric Research, Hobart, Tasmania, Australia; Technical University of Denmark, Denmark

## Abstract

Spatial variation in growth is a common feature of demersal fish populations which often exist as discrete adult sub-populations linked by a pelagic larval stage. However, it remains unclear whether variation in growth occurs at similar spatial scales for populations of highly migratory pelagic species, such as tuna. We examined spatial variation in growth of albacore *Thunnus alalunga* across 90° of longitude in the South Pacific Ocean from the east coast of Australia to the Pitcairn Islands. Using length-at-age data from a validated ageing method we found evidence for significant variation in length-at-age and growth parameters (*L*_∞_ and *k*) between sexes and across longitudes. Growth trajectories were similar between sexes up until four years of age, after which the length-at-age for males was, on average, greater than that for females. Males reached an average maximum size more than 8 cm larger than females. Length-at-age and growth parameters were consistently greater at more easterly longitudes than at westerly longitudes for both females and males. Our results provide strong evidence that finer spatial structure exists within the South Pacific albacore stock and raises the question of whether the scale of their “highly migratory” nature should be re-assessed. Future stock assessment models for South Pacific albacore should consider sex-specific growth curves and spatial variation in growth within the stock.

## Introduction

Knowledge of the growth of individuals is essential for understanding the processes shaping populations and for managing exploited fish species [1]. Growth estimates are typically derived from information on the size and age of individuals or from tag-recapture studies [2]. Collecting sufficient data across an entire population to obtain reliable estimates of growth can often be difficult and expensive. Therefore, estimates of growth are commonly drawn from a single location or averaged across multiple locations, without consideration of spatial variation in growth [3], [4]. However, spatial variation in growth is likely to have significant implications for population dynamics [5], defining population structure for assessment models, and delineating management units [4].

Variation in growth between populations can result from genotypic variation or from plastic phenotypic responses to variation in local environmental factors such as temperature and food availability [6]. Spatial variation in growth is a common feature of demersal fish populations and has been observed at multiple scales ranging from patch reefs on the same reef (<1 km) [7], [8] to thousands of kilometres across an ocean basin [9], [10]. Many demersal species display a metapopulation structure [11], with spatial separation of adult sub-populations linked to varying degrees by a pelagic larval stage, and spatial variation in growth is expected. However, the metapopulation paradigm may not be applicable to highly migratory pelagic species such as tunas which typically do not have a strong association with benthic habitat and are assumed to exhibit a continuous distribution as a result of high mobility as adults. There have been no comprehensive studies of spatial variation in growth within a stock for large pelagic species, although [12] demonstrated a difference in growth of bigeye tuna (*Thunnus obesus*) between stocks in the Pacific and Indian Oceans. Therefore, it remains unclear whether the spatial variation in growth observed for many demersal species is evident in stocks of migratory pelagic species such as tuna that are assumed to move at the scale of ocean basins.

Albacore tuna (*Thunnus alalunga*) are widely distributed in all three oceans between approximately 50° N and 40° S, although their abundance is relatively low in equatorial areas [13]. There are at least six genetically distinct stocks of albacore, located in the North and South Pacific Ocean, North and South Atlantic Ocean, the Indian Ocean and the Mediterranean Sea [14], [15], [16], [17], [18]. Albacore are a moderate-sized tuna with a maximum reported size of 127 cm fork length (*FL*) and 40 kg [13]. Albacore is a commercially important species contributing 6% to the annual global tuna catch of 4.38 million tonnes in 2009 [19]. One of the largest fisheries for albacore is in the South Pacific Ocean where annual harvest peaked at more than 80,000 mt in 2010, driven mainly by increased longline catches [20]. Albacore have been commercially harvested throughout their distribution in the South Pacific, but most of the catch is taken between 10 and 30° S and 160°E and 160° W [21].

**Figure 1:**
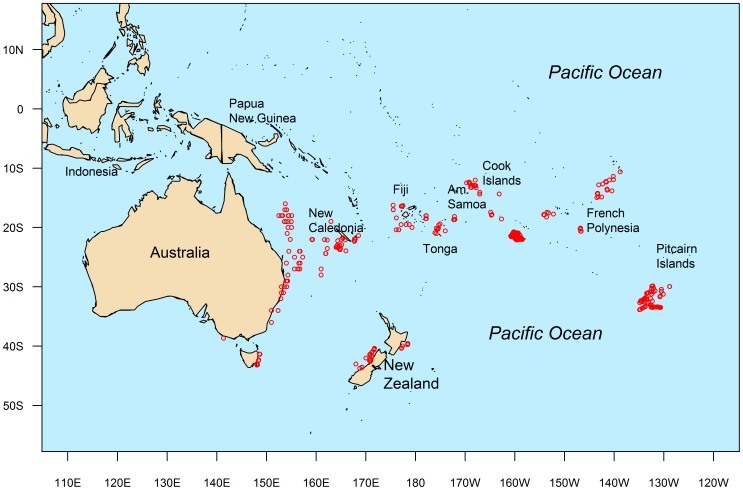
Map indicating locations where samples were collected.

In the South Pacific, mature albacore (>80 cm *FL*) spawn in tropical and sub-tropical waters between about 10° S and 25° S during the austral summer [22], [23], with juveniles recruiting to temperate waters in the vicinity of the sub-tropical convergence zone (∼40° S) about one year later, at a size of 45−50 cm *FL*. Tagging data indicate that juvenile albacore gradually disperse to the north from these southern latitudes [24], while catch rate data suggest that larger albacore make seasonal migrations between tropical and sub-tropical waters [25]. The consequence of these movement patterns is a latitudinal gradient in size distribution, with predominantly small fish (<80 cm *FL*) at high latitudes (south of 35° S) and large fish (>80 cm *FL*) at lower latitudes (north of 30° S).

**Table 1:** Number of albacore from which age estimates were obtained for male, female and unknown sex individuals in each region of the South Pacific.

Region	Female	Male	Unknown sex	Total
American Samoa	72	122		194
Australia	455	219	4	678
Cook Islands	41	111		152
Fiji	15	92	1	108
French Polynesia	103	123		226
International Waters 1	49	11		60
International Waters 2	8	9	55	72
New Caledonia	85	105	7	197
New Zealand	89	82	3	174
Tonga	53	55		108
Total	970	929	70	1969

International Waters 1 refers to the waters between the Australian and New Caledonian EEZs, International Waters 2 refers to the waters south of the Pitcairn Islands.

There have been numerous studies of albacore growth throughout their distribution, most of which have estimated growth parameters from modal analysis of length frequency data, tag-recapture experiments or age estimates from scales, vertebrae or spines (e.g. [26], [27], [28], [29], [30]). Very few studies have used increment counts in otoliths to estimate age and growth of albacore over their entire lifespan, perhaps due to the perceived difficulty of interpreting annual increments in tuna otoliths. More recently, however, otoliths have been shown to be a reliable structure for estimating age for large pelagic species, with several studies validating techniques for estimating the age of tuna, including South Pacific albacore, from counts of annual increments [12], [23], [31], [32], [33]. These studies have provided a foundation for the use of otoliths to estimate age-based population parameters for tropical and temperate tuna.

In this study, we examine variation in growth of albacore across 90 degrees of longitude, from the east coast of Australia (140°E) to the Pitcairn Islands (130° W), in the South Pacific Ocean. This encapsulates much of the range of albacore in the South Pacific. Given the strong evidence of an annual north-south migration of albacore [25], an explicit analysis of latitudinal patterns in growth was not considered. We use an information theoretic model selection procedure to determine the most appropriate growth model to fit to annual increment data from validated ageing techniques and to assess whether there are differences in growth between female and male albacore. Finally, we fit growth models with longitudinal terms to estimate the level of longitudinal variation in length-at-age and growth parameters for both sexes.

**Figure 2:**
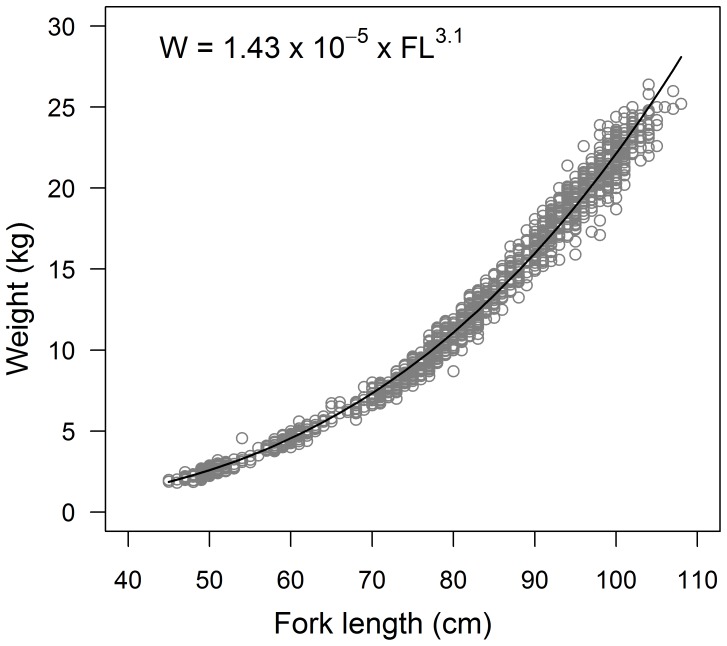
Weight-at-length data and fitted power curve for South Pacific albacore (n = 1756).

## Methods

### Sample Collection

Ethical approval was not required for this study, as all fish were collected as part of routine fishing procedures. No samples were collected by the authors. All samples in this study originated from commercial or recreational fisheries (New Zealand commercial Albacore Fishery, Western Central Pacific Ocean commercial longline fishery, and Australian commercial Eastern Tuna and Billfish Fishery [ETBF] and recreational fishery) and were already dead when provided to the sampler. Fish were sacrificed by the commercial or recreational fisher at sea using standard fisheries practices (most fish were dead when landed). Permission was granted to use samples from all fish. All samples were donated.

**Table 2:** Parameter estimates (± standard error) from five candidate growth models for South Pacific albacore.

Sex	Model	*L*_∞_	*k*	*t*	*p*	*δ*	*γ*	*V*	AIC_c_	ΔAIC_c_	*w*
All	VBGM	104.52 (0.44)	0.40 (0.01)	−0.49 (0.05)					11831.67	23.89	0
	Gompertz	103.09 (0.37)	0.50 (0.01)	0.47 (0.03)					11811.54	3.77	0.08
	Logistic	102.09 (0.33)	0.61 (0.01)	1.12 (0.03)					11807.77	0.00	0.53
	Richards	102.30 (0.49)	0.58 (0.04)	0.98 (0.24)	1.32 (0.68)				11809.40	1.63	0.24
	Schnute-Richards	101.52 (0.60)	0.05 (0.08)			−0.97 (0.08)	3.54 (2.65)	2.07 (0.76)	11810.25	2.48	0.15
Female	Logistic	96.97 (0.37)	0.69 (0.02)	0.99 (0.03)					5746.90		
Male	Logistic	105.34 (0.44)	0.59 (0.02)	1.25 (0.04)					5729.26		

AIC_c_ is the small-sample bias-corrected form of Akaike’s information criterion, Δ_i_ is the Akaike difference, and *w*_i_ is the Akaike weight. Note that the parameters *k* and *t* are defined differently in each model (see text for definitions), such that values are not comparable across m.

No field permits were required to collect any samples from any location, as all samples originated from commercial and recreational catch. Albacore tuna are not a protected species in any ocean.

**Figure 3:**
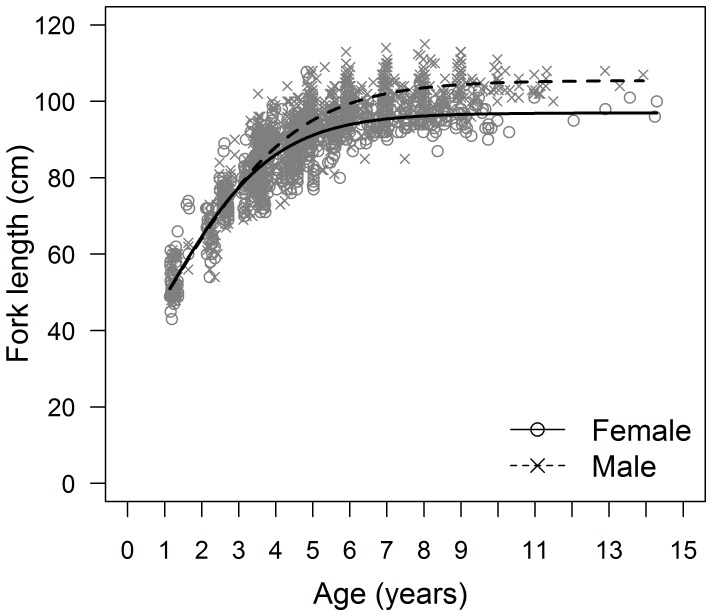
Length-at-age data and logistic growth models for female and male South Pacific albacore.

Sagittal otoliths and gonads were collected from 3082 albacore sampled from the South Pacific Ocean between January 2009 and December 2010. Fish were caught from an area between 130°E to 130° W from waters off Australia, New Zealand, New Caledonia, Fiji, Tonga, American Samoa, Cook Islands, French Polynesia, and in a region south of the Pitcairn Islands (Fig. 1). Within Australia, fish were sampled from the ETBF and the recreational fishery in ports along the east coast. Fish sampled in the ETBF were caught between ∼14° S and 37° S, while fish sampled from the recreational fishery were caught between 37° S and 44° S. In New Zealand, albacore were sampled south of 35° S from the domestic troll fishery and also during chartered tagging operations. Samples collected from all other regions were collected mostly north of 25° S (except for south of the Pitcairn Islands) either by observers on longline fishing vessels or directly by the fishing crew of longline fishing vessels.

Otoliths and gonads were removed, frozen and sent to the laboratory where they were cleaned of residual material, dried and archived. Fork length (*FL*) was measured to the nearest cm for all fish, except those from south of the Pitcairn Islands for which reliable length data were unavailable. Accurate weight data were available for these fish, however, so the relationship between whole weight and *FL* was used to estimate *FL* for these individuals. Whole weight (*W*) was measured to the nearest 0.1 kg for most fish sampled in Australia, New Zealand, and south of the Pitcairn Islands. Sex was identified for most fish based on macroscopic or histological examination of the gonads as described by [23]. Those fish for which sex was not determined were excluded from further analyses.

**Table 3:** Parameter estimates from linear mixed effects (LME) and generalized linear models (GLM).

Sex	Model type	Longitudinal effect	Model	AIC_c_	ΔAIC_c_	*w*
Female	LME	None	*R* = *β*_set_+*ε*	5677.34	75.93	0
		Linear	*R* = *α*_lon_+*β*_set_+*ε*	5616.06	14.65	0
		Cubic spline (df = 2)	*R* = *spline*_2_(*lon*)+*β*_set_+*ε*	5602.34	0.93	0.39
		Cubic spline (df = 3)	*R* = *spline*_3_(*lon*)+*β*_set_+*ε*	5601.41	0	0.61
	GLM	Linear	*R* = *α*_lon_+*ε*	5627.45	4.01	0.07
		Cubic spline (df = 2)	*R* = *spline*_2_(*lon*)+*ε*	5623.44	0	0.51
		Cubic spline (df = 3)	*R* = *spline*_3_(*lon*)+*ε*	5623.83	0.39	0.42
Male	LME	None	*R* = *β*_set_+*ε*	5667.88	129.33	0
		Linear	*R* = *α*_lon_+*β*_set_+*ε*	5562.72	24.17	0
		Cubic spline (df = 2)	*R* = *spline*_2_(*lon*)+*β*_set_+*ε*	5547.01	11.48	0
		Cubic spline (df = 3)	*R* = *spline*_3_(*lon*)+*β*_set_+*ε*	5535.06	0	1.00
	GLM	Linear	*R* = *α*_lon_+*ε*	5561.54	10.87	0
		Cubic spline (df = 2)	*R* = *spline*_2_(*lon*)+*ε*	5559.78	9.12	0.01
		Cubic spline (df = 3)	*R* = *spline*_3_(*lon*)+*ε*	5550.67	0	0.99

*R* is the residual fork length, *α*_lon_ is the effect of longitude (lon), *β*_set_ is the random effect of fishing set, and *ε* is the error term. AIC_c_ is the small-sample bias-corrected form of Akaike’s information criterion, Δ_i_ is the Akaike difference, and *w*_i_ is the Akaike weight.

**Figure 4:**
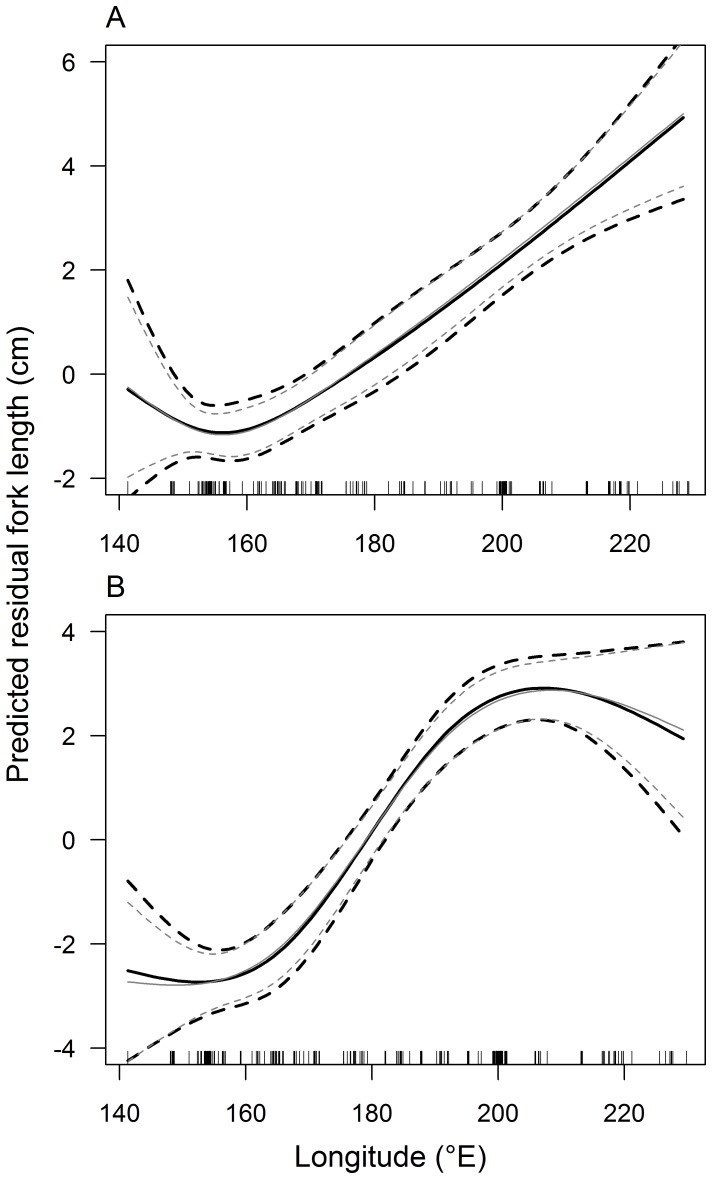
Predicted longitudinal trends in residual fork lengths of female (A) and male (B) South Pacific albacore. Predictions from cubic splines with 3 degrees of freedom are shown for LMEs (dark lines) and GLMs (light lines). Dashed lines represent 2 standard deviations from the mean.

### Age Estimation

A subsample of otoliths were selected for age estimation based on sampling location, *FL* and sex with the aim of estimating age for the full size range of females and males caught in each region. Age estimates were obtained directly from the study by [23], who used validated methods to count increments in sectioned otoliths and estimate age in decimal years. Age estimates were available for 1969 albacore and sex information was available for 1899 of these fish (Table 1).

### Length-weight Relationship

The relationship between *FL* and *W* was estimated using a power function of the form 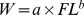 where *a* is the coefficient of the power function and *b* is the exponent indicating isometric growth when equal to 3. Weight-at-length data were available only from Australia and New Zealand, although the size range of fish from New Zealand was limited mostly to individuals <70 cm FL. Therefore, the *FL*-*W* relationship was compared between males and females within Australia and New Zealand using an analysis of covariance (ANCOVA) with *FL* as the covariate of *W*. A second ANCOVA was then used to compare the *FL*-*W* relationship between Australia and New Zealand using a common length-range for each region. Length and weight data were log-transformed for the analysis to satisfy the assumption of linearity.

**Table 4:** Summary of growth models used to examine longitudinal variation in growth parameters *k* and *L_∞_*.

Longitude functions			Female			Male		
*f*_1_	*f_2_*	*K*	AIC_c_	ΔAIC_c_	*w*	AIC_c_	ΔAIC_c_	*w*
*L*_∞_	*k*	4	5746.90	140.76	0	5729.26	224.51	0
*L*_∞+_ *α*_1_ *l*	*k*	5	5618.36	12.22	0	5532.11	27.36	0
*L*_∞_+*α*_1_ *l*+*α*_2_ *l* ^2^	*k*	6	5618.49	12.35	0	5519.44	14.69	0
*L*_∞_	*k*+*β*_1_ *l*	4	5623.76	17.61	0	5522.68	17.93	0
*L*_∞_	*k*+*β*_1_ *l*+*β*_2_ *l* ^2^	5	5608.20	2.06	0.16	5524.52	19.76	0
*L*_∞_+*α*_1_ *l*	*k*+*β*_1_ *l*	6	5611.47	5.32	0.03	5516.91	12.16	0
*L*_∞_+*α*_1_ *l*+*α*_2_ *l* ^2^	*k*+*β*_1_ *l*	7	5609.80	3.65	0.07	5507.89	3.14	0.17
*L*_∞_+*α*_1_ *l*	*k*+*β*_1_ *l*+*β*_2_ *l* ^2^	7	5606.14	0	0.44	5513.20	8.45	0.01
*L*_∞_+*α*_1_ *l*+*α*_2_ *l* ^2^	*k*+*β*_1_ *l*+*β*_2_ *l* ^2^	8	5606.94	0.80	0.30	5504.75	0	0.82

*f*_1_ and *f*_2_ are functions of the growth parameters where α_1_ and α_2_ describe the relationship between *L_∞_* and *l*, and *β*_1_ and *β*_2_ describe the relationship between *k* and *l*. *K* is the number of estimated model parameters (plus one for variance). AIC_c_ is the small-sample bias-corrected form of Akaike’s information criterion, Δ_i_ is the Akaike difference, and *w*_i_ is the Akaike weight.

**Figure 5:**
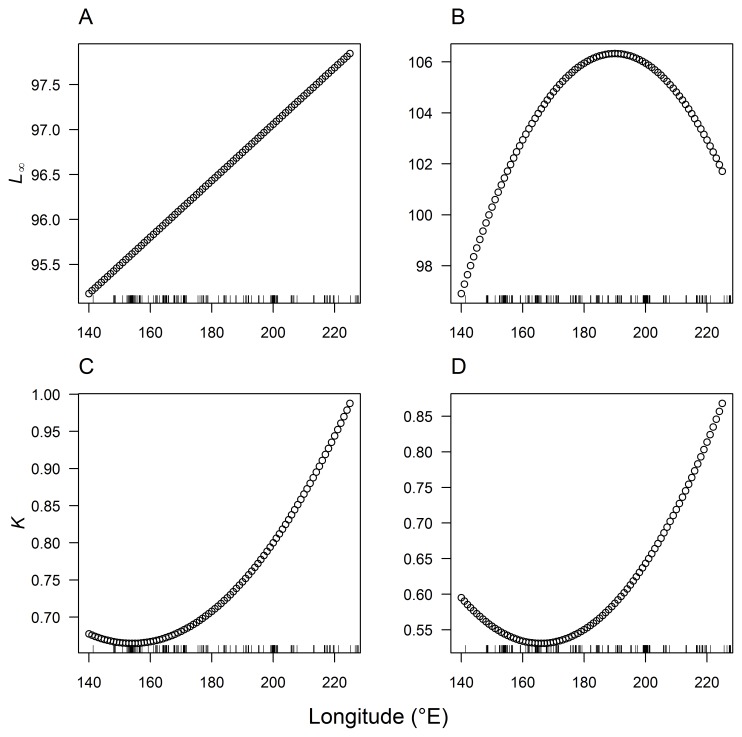
Predicted longitudinal trends in female (A and C) and male (B and D) growth model parameters *L*_∞_ and *k*.

**Table 5:** Predicted logistic growth model parameter estimates in the west (150°E), central (185°E) and east (220°E) South Pacific Ocean based on non-linear variation in *k* and linear variation in *L*_∞_ for females, and non-linear variation in *k* and *L*_∞_ for males.

Sex	Region	*L*_∞_ (cm)	*k*	*t*_0_ (years)
Female	West	95.49	0.67	0.92
	Central	96.59	0.73	0.92
	East	97.69	0.94	0.92
Male	West	100.30	0.56	1.06
	Central	106.23	0.57	1.06
	East	102.93	0.81	1.06

Common values of the growth parameter *t_0_* were used across regions for males and females.

**Figure 6:**
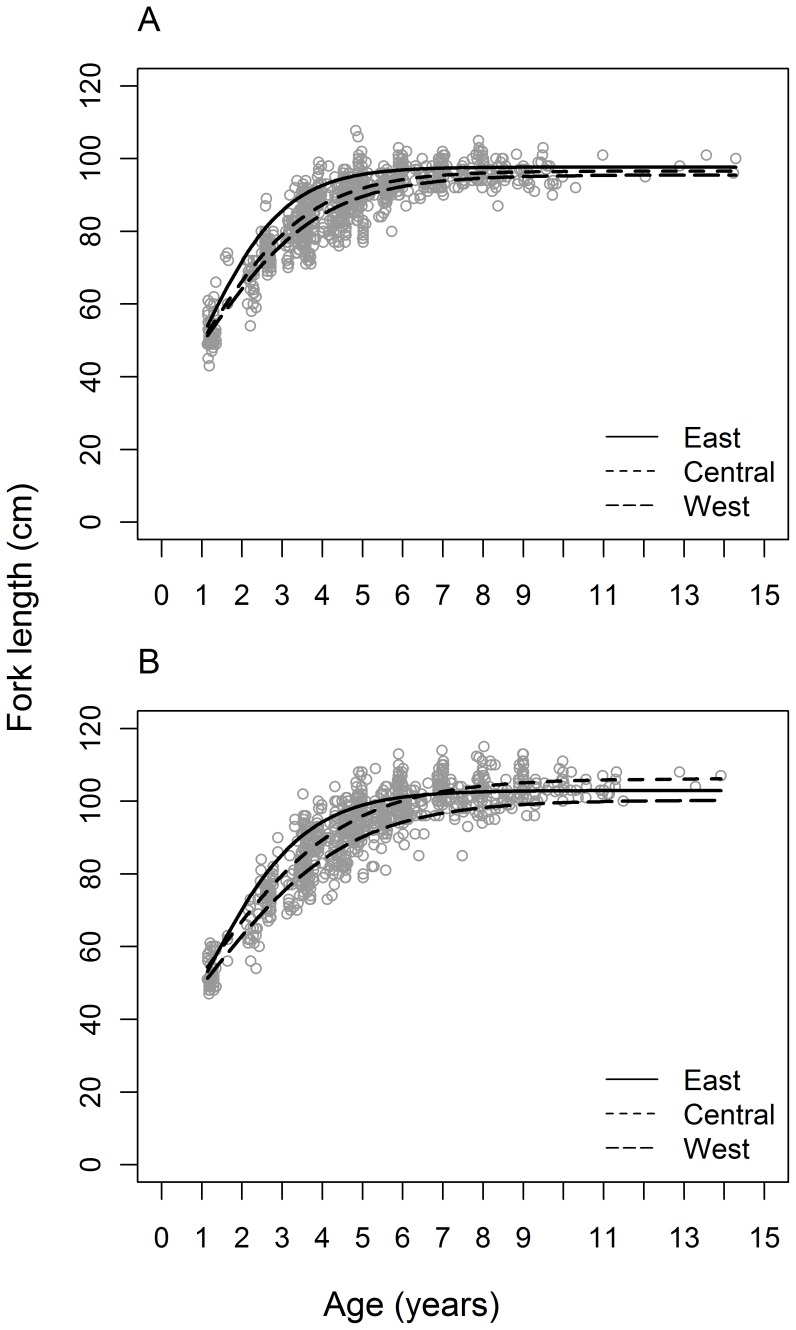
Predicted growth curves in the west (150°E), central (185°E) and east (220°E) South Pacific Ocean.

### Growth

We used an information theoretic, multi-model inference approach to determine the optimal growth model for albacore [34]. We fitted length-at-age data to a set of five candidate models commonly used for teleosts which included the von Bertalanffy (VBGM) [35], Gompertz [36], Logistic [37] and Richards [38], and the generalised growth model proposed by [39] of which all the other models are special cases. The form of the VBGM was:
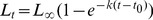
(1)where *L_t_* is the fork length at age *t*, *L_∞_* is the mean asymptotic length, *k* is a relative growth rate parameter (year^−1^), and *t*_0_ is the age at which fish have a theoretical length of zero.

The Gompertz and Logistic models are both three parameter sigmoidal curves that assume that the growth rate decreases exponentially with size. They typically characterize growth well where growth is relatively slow early in life [33]. The form of these models was:
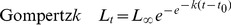
(2)

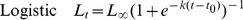
(3)where *L_∞_* is the mean asymptotic length, *k* is the rate of exponential decrease of the relative growth rate with age (year^−1^), and *t_0_* is the age at the inflection point of the curve.

The four parameter sigmoidal Richards model is equivalent to the generalised VBGM proposed by [40] and the form used here was:
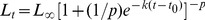
(4)where *L_∞_* is the mean asymptotic length, *k* is a relative growth rate parameter (year^−1^), *t_0_* is the age at the inflection point of the curve, and *p* is a dimensionless parameter.

The five parameter Schnute-Richards was proposed as an omnibus approach to modelling fish growth and is useful for modelling growth where the relationship between length and age is allometric [41]. The form of the Schnute-Richards model was:
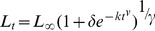
(5)where *L_∞_* is the mean asymptotic length, *k* is a relative growth rate parameter (year^-*v*^) and *δ*, *γ* and *v* are dimensionless parameters for which particular values provide special cases equivalent to models (1) to (4).

The five candidate models were fitted to albacore length-at-age data using non-linear least squares in R version 2.13.2 [42]. We evaluated the relative support for each model using Akaike’s Information Criteria for small sample sizes (AIC_c_: [43]). Models with an AIC_c_ value within two of that calculated for the best approximating model (lowest AIC_c_) were considered to describe the data equivalently well [43]. The Akaike weight, *w_i_* [43], of each model *i* was calculated to quantify the plausibility of each model, given the data and the set of five models using:
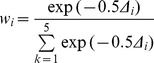
(6)where Δ*_i_* = AIC_c,min_–AIC_c,*i*_. The Akaike weight is considered as the weight of evidence in favour of model *i* being the actual best model of the available set of models.

We evaluated support for sex-specific growth curves by comparing the AIC_c_ from the best fit model for all data to the sum of the AIC_c_s from the same model fitted separately to male and female data. This comparison indicated substantial support for separate growth curves for females and males. Accordingly, separate growth models for females and males were used to evaluate support for longitudinal variation in albacore growth across the South Pacific Ocean.

Evidence for longitudinal variation in length-at-age was examined using generalised linear models (GLM) and linear mixed-effects models (LME) to model the effects of longitude on the residual length-at-age data from the growth models for males and females. The relationship between the residuals and longitude was explored by modelling longitude as a linear and non-linear (cubic spline with 2 or 3 degrees of freedom) variable in both the GLM and LME. AIC_c_ was used to determine whether there was support for including longitude in the models and, if so, which functional form for the relationship between the residuals and longitude was best supported by the data. Fishing set was modelled as a random effects term in the LME models because multiple individual fish were often sampled at the same time from a single location and, therefore, not all samples were independent. Results from LMEs and GLMs were compared graphically to evaluate the effects of the non-independent sampling. To determine whether samples collected at higher latitudes from New Zealand and Australia confounded the longitudinal analyses for all data, the same analyses were conducted on a subset of the length-at-age data from latitudes north of 25°S.

Longitudinal variation in the pattern of albacore growth was explored by including additional parameters in the growth models for females and males. The growth parameters *L_∞_* and *k* were expected to be more affected by longitude than other growth parameters. Accordingly, *L_∞_* and *k* were modelled as linear or quadratic functions of longitude in the best-fit growth models for females and males, and fitted to length-at age data using least squares. AIC_c_ and Akaike weights were used to determine whether there was support for including longitude in the growth models and, if so, which functional form for the relationship between each growth parameter and longitude was best supported by the data. To estimate the magnitude of variation in growth of albacore that could be expected across the longitudinal range of samples collected in this study, we calculated the predicted growth parameters from the best-fit growth models that incorporated parameters for longitude, and plotted the resulting growth curves, for the central (185°E), west (150°E) and east (220°E) longitudes of the study area.

## Results

The relationship between *FL* and *W* did not differ significantly between male and female albacore in Australia (*F* = 3.32, df = 1, p = 0.07) or New Zealand (*F* = 0.92, df = 1, p = 0.34), or for sexes combined between Australia and New Zealand (*F* = 3.90, df = 1, p = 0.05), so data were pooled across sexes and regions (Fig. 2). The estimated parameters and 95% confidence intervals of the *FL*–*W* relationship (*a* = (1.43±0.87)×10^−5^, *b* = 3.10±0.01) indicate that albacore exhibit a positively allometric growth pattern (*b*>3) in which their girth increases disproportionately to length.

None of the five candidate growth models was unambiguously the best model for albacore growth as indicated by ΔAIC_c_ values <3 and Akaike weights between 0.15 and 0.53 for the three best-fitting growth models (Table 2). The logistic model was found to be the best approximating model among all candidate models (*w* = 0.53), although there was substantial support also for the Richards model (ΔAIC_c_ = 1.63, *w* = 0.24). There was less support for the Schnute-Richards and Gompertz models (ΔAIC_c_ values >2) and the VBGM was least supported among the set of candidate models with a ΔAIC_c_ >10 and weight of evidence of 0.

Separate sex-specific growth models were strongly supported by the data with a difference of 331.61 between the AIC_c_ from the best fit of the logistic model to all data (AIC_c_ = 11807.77) and the sum of the AIC_c_s from the fit of the same model to female and male data (AIC_c_ = 5746.90+5729.26 = 11476.16) (Table 2). The fitted logistic growth curves for females and males were very similar up until age 4 years, after which the length-at-age for males was on average greater than that for females (Fig. 3). The predicted mean asymptotic length (*L_∞_*) from the best-fit models was 8.37 cm greater for males than for females (Table 2).

The LME and GLM models revealed that the residual length-at-age from the logistic growth models for females and males varied significantly with longitude (Table 3). The relationship between residual length-at-age and longitude was described best by a cubic spline with 3 degrees of freedom for both females and males, as indicated by the lowest AIC_c_ values for these models (Table 3). However, there was also substantial support (ΔAIC_c_ = 0.93, *w* = 0.39) for a cubic spline model with 2 degrees of freedom for females. There was a strong relationship between the residuals and longitude for both males and females, with residual length-at-age predicted to be consistently greater at more easterly longitudes than at westerly longitudes (Fig. 4). The results from the GLM and LME models were nearly identical (Fig. 4) indicating a negligible effect from the non-independent samples, which were not considered in subsequent analyses. Very similar results were obtained from an analysis of a subset of the length-at-age data from latitudes north of 25° S, suggesting that the observed longitudinal patterns in the residuals were not affected significantly by latitude or by the different fishing gears used (longline and troll). Similarly, adding a latitude term to the regression did not improve model fit.

The inclusion of additional parameters for longitude in the growth models was strongly supported by both female and male length-at-age data (Table 4). A growth model with non-linear variation in *k* and linear variation in *L_∞_* was best supported (*w* = 0.44) by the data for females, although there was also substantial support (ΔAIC_c_ = 0.80, *w* = 0.30) for a growth model with non-linear variation in *k* and *L_∞_* (Table 4). For males, there was unequivocal support for a growth model with non-linear variation in *k* and *L_∞_* with a weight of evidence of 0.82. In all cases, growth models predicted that *k* and *L_∞_* were larger at more easterly longitudes than at westerly longitudes for both females and males (Fig. 5), similar to the trends in fork length residuals (Fig. 4). However, predicted values of *L_∞_* peaked at around 190°E for males, and the lowest values of *k* were predicted at approximately 155°E for females and 165°E for males (Fig. 5). The predicted magnitude of variation in *k* across longitudes was similar for females and males (approx. 55%), but the predicted magnitude of longitudinal variation in *L_∞_* was greater for males (9%) than for females (3%) (Fig. 5). Predicted growth curves in the west (150°E), central (185°E) and east (220°E) regions of the South Pacific demonstrate the magnitude of variation in growth of albacore that could be expected across the longitudinal range of this study (Table 5, Fig. 6). These growth curves show the small variation in *L_∞_* and larger variation in *k* for female albacore, with higher *k* values in the east than in the central and west regions. For males, these growth curves demonstrate the variation in *L_∞_* and *k*, with lower *L_∞_* and *k* values in the west region than in the central and east regions.

## Discussion

We found evidence for significant variation in length-at-age of albacore between females and males and across 90 degrees of longitude in the South Pacific Ocean. Growth varied more between sexes than with longitude, with males growing to an average maximum size nearly 10% greater than females. The magnitude of longitudinal variation in length-at-age was similar for females and males, with fish approximately 6 cm longer on average at eastern longitudes than at western longitudes. However, longitudinal variation in average maximum size was more pronounced for males than for females, with the greatest difference in average maximum size being 2% and 6% for females and males, respectively. The magnitude of spatial variation observed for South Pacific albacore was comparable to that observed for other demersal marine species at similar spatial scales (e.g. [9], [10], [44], suggesting that the forces structuring local adaptation in growth can operate at similar spatial scales for demersal and highly mobile pelagic species.

The observed longitudinal variation in albacore length-at-age could be explained by spatial variation in the selectivity of fishing gear, variable availability due to size-specific migration, or spatial variation in growth. Small albacore decline significantly as a proportion of the longline catch at more easterly longitudes, compared to the west [45], which suggests that selectivity or availability may influence observed length-at-age. Selection for larger individuals at eastern longitudes would result in larger observed length-at-age in the catch (but not the population) in the east. Similar longline gear is used throughout the fishery, so spatial variation in selectivity may be less likely, or make less contribution, than the effects of availability or spatial variation in growth. Net migration of larger individuals from western to more easterly longitudes may also result in a larger mean length-at-age in the east. However, there are no tagging data available to support or refute size-specific migration, so the contribution of size-specific movement to the observed variation in length-at-age is unknown. Nevertheless, many implications of the observed longitudinal variation in growth of albacore remain equally relevant whether they result from the migration of individuals or reflect spatial differences in local growth.

Spatial variation in growth of fishes may result from variation in environmental factors, genetics, or a combination of both [46]. Separating the effects of phenotypic and genotypic variation is difficult and ‘common garden’ experiments are usually employed to control environmental variables so as to reveal the environmental and genetic components of phenotypic variation [46]. Such experiments are possible for small sedentary species, but impractical for large highly mobile pelagic species such as tuna. It will be difficult, therefore, to assess the relative contribution of environmental and genetic variation to the observed spatial variation in growth of albacore.

Growth in fishes often varies spatially with environmental gradients in abiotic or biotic factors, such as temperature and food availability [6]. Water temperature affects growth in many fish populations [47]. Often, fish populations with a broad latitudinal range grow faster and larger at higher latitudes, where water temperatures are cooler [46], [47], [48], [49], [50], [51]. However, this pattern has generally been observed for species in which individuals remain in a similar environment. The effects of temperature and other oceanographic variables on the growth of migratory species such as albacore are less clear. Albacore experience water temperatures ranging from at least 8 to 30°C during their seasonal migrations and vertical movements in the water column [52], [53], although adults in the South Pacific appear to prefer a temperature range between 20 and 25°C [52]. Temperature and other oceanographic variables such as salinity and dissolved oxygen vary considerably across the Pacific Ocean, but there are no obvious longitudinal patterns consistent with the observed variation in growth [54]. Therefore, it remains unclear how variation in temperature or other oceanographic variables may affect growth of albacore in the South Pacific.

Food availability is well known to directly affect fish growth [6], with numerous studies demonstrating a positive correlation between availability of food and growth [55], [56], [57]. Preliminary diet studies of albacore in the South Pacific Ocean indicate that fish (e.g. Paralepidae and Myctophidae) are the dominant prey, followed by squids and crustaceans [58]. The distribution and abundance of these taxa in the South Pacific Ocean remains unknown, but it is generally considered that micronekton densities are higher at ocean fronts and eddies [59], [60] where growth rates of predators are enhanced [61]. The spatial and temporal distribution of ocean fronts and eddies in the western and central South Pacific Ocean is complex, with numerous areas of convergence and enhanced eddy activity [62]. However, there is no evidence for increased fronts or eddy activity at the longitudes where the length-at-age of albacore was greatest. Information on the horizontal and vertical distribution of albacore prey across the region is required to examine potential linkages between albacore growth rates and the availability of forage. The integration of data from diet studies, archival tagging and stable isotope analyses of soft tissues would provide such information and allow a more comprehensive evaluation of the effects of prey availability on albacore growth.

The growth trajectories for female and male South Pacific albacore were similar up until 4 years of age, after which the length-at-age for males was, on average, greater than that for females. The divergence in growth trajectories is most likely due to the onset of female maturity, which occurs at approximately 80–85 cm *FL* [23], and the subsequent additional energy required for ovarian development compared with spermatogenesis. A similar pattern was observed for southern bluefin tuna (*Thunnus maccoyii*) in the Indian Ocean where female growth slowed relative to males after reaching the size at maturity [63]. Our results are consistent with other growth studies of albacore that have demonstrated significant differences in growth between females and males in the North Atlantic Ocean [29] and Mediterranean Sea [28]. However, our results contrast those from [27] who found no significant difference in growth between female and male South Pacific albacore using age estimates from increment counts in vertebrae. The use of vertebrae to estimate age has resulted in significant underestimates of age in other species, including other tunas [64], suggesting that the growth curves estimated by [27] are likely negatively biased. Furthermore, their sample size of females (59) and males (70) was relatively low, potentially resulting in bias and imprecision in estimates of *L*_∞_ and *k* and low power to detect differences in growth between sexes.

Outputs from stock assessments for South Pacific albacore are highly sensitive to structural assumptions about growth and input estimates of growth parameters [65], [66]. The current assessment model assumes a single growth curve for the entire South Pacific stock, which is estimated by fitting a VBGF model to length frequency data [66]. Our results provide an opportunity to refine and improve the structure of the assessment model by assimilating into the model a better depiction of growth patterns for South Pacific albacore. Firstly, our results strongly support the inclusion of separate growth curves for females and males. The size-at-age of females (>4 years) will be overestimated using a single growth curve for both sexes, potentially biasing other parameters estimated from the growth curve, such as maturity, spawning stock biomass and related management reference points. Secondly, the significant longitudinal variation in observed length-at-age may contradict the assumption of homogeneity in growth within the South Pacific albacore stock, and suggests that the use of a single growth curve for the entire stock may not be appropriate, even if sex-specific growth is accommodated.

This is the first study to examine explicitly the spatial variation in growth across an entire stock for any tuna species. We have demonstrated substantial differences in growth of albacore between sexes and in observed length-at-age across 90 degrees of longitude in the South Pacific Ocean. The causes of the longitudinal variation in growth of albacore remain unclear, but future studies that integrate diet information with archival tagging and stable isotope analyses may provide some insights into the potential linkages between prey availability and albacore growth. Future development of assessment models for South Pacific albacore is likely to benefit from explicit consideration of sex-specific growth curves and, potentially, variation in growth within the stock. Such structural improvements are likely to provide more reliable estimates of biomass, fishing mortality and potential yields, and ultimately provide the foundation for more robust management decisions.
